# Blood perfusion with polymyxin B immobilized columns in patients with COVID-19 requiring oxygen therapy

**DOI:** 10.1038/s41598-024-63330-2

**Published:** 2024-05-31

**Authors:** Daisuke Katagiri, Akinari Tsukada, Shinyu Izumi, Yosuke Shimizu, Junko Terada-Hirashima, Yukari Uemura, Yusaku Kusaba, Jin Takasaki, Hiroyuki Takoi, Miwa Tamura-Nakano, Masayuki Hojo, Hideki Takano, Eisei Noiri, Shinji Abe, Arata Azuma, Haruhito Sugiyama

**Affiliations:** 1 Department of Nephrology, Center Hospital of the National Center for Global Health and Medicine, Tokyo, Japan; 2 Department of Respiratory Medicine, Center Hospital of the National Center for Global Health and Medicine, Tokyo, Japan; 3 Center for Clinical Sciences, National Center for Global Health and Medicine, Tokyo, Japan; 4 Department of Respiratory Medicine, Tokyo Medical University Hospital, Tokyo, Japan; 5 Communal Laboratory, Research Institute, National Center for Global Health and Medicine, Tokyo, Japan; 6 National Center Biobank Network, National Center for Global Health and Medicine, Tokyo, Japan; 7 Department of Pulmonary Medicine and Oncology, Graduate School of Medicine, Nippon Medical School, Tokyo, Japan; 8 Mihara General Hospital, Pulmonary Medicine and Clinical Research Center, Saitama, Japan

**Keywords:** Viral infection, Respiratory distress syndrome

## Abstract

Extracorporeal blood purification with polymyxin B immobilized fiber column direct hemoperfusion (PMX-DHP), is reported to be effective in treating COVID-19 pneumonitis with oxygen demand. This multicenter prospective study evaluated the efficacy and safety of PMX-DHP in oxygen-requiring patients with COVID-19 admitted between September 28, 2020, and March 31, 2022. The primary endpoint was the percentage of clinical improvement 15 days after treatment. The secondary endpoint was the percentage of worsened disease status. Data from the COVID-19 patient registry were used for the synthetic control group. The improvement rate on Day 15 did not differ between PMX-treated patients and controls; however, the deterioration rate was 0.38 times lower in the PMX-treated group, and the death rates on Day 29 were 0 and 11.1% in the PMX-treated and control groups, respectively. The PMX group showed a 0.73 times higher likelihood for reduced intensive care demand, as 16.7% of PMX-treated patients and 22.8% of controls worsened. After treatment blood oxygenation improved, urinary β2-microglobulin and liver-type fatty acid-binding protein showed significant decreases, and IL-6 decreased once during treatment but did not persist. In this study, PMX treatment effectively prevented the worsening of COVID-19 pathology, accompanied by improved oxygenation. PMX treatment to remove activated cells may effectively improve patient outcomes.

## Introduction

Coronavirus disease 2019 (COVID-19) quickly spread worldwide and caused extensive damage after an outbreak of pneumonia of unknown cause was reported in China in December 2019. Numerous epidemics have emerged leading to the global promotion of vaccination that demonstrated remarkable effectiveness. Notably, when multiple doses are administered, infected patients are often asymptomatic. However, some unvaccinated patients, or those who are at high risk for severe disease, require intensive care such as ventilatory management or extracorporeal membrane oxygenation. The virus causes microvascular COVID-19 lung vessels obstructive thrombo-inflammation syndrome^1^. Importantly, the target organs are not limited to the lungs: COVID-19 is a systemic disease that causes increased thrombus formation, embolization, and bleeding, either through direct effects on platelets and vascular endothelium or indirectly by impaired inflammation and coagulation^2^.

Patients with cardiovascular risk factors, such as diabetes and obesity, are more prone to severe disease due to thrombosis, endothelial damage, and chronic inflammation. Combination therapies targeting these symptoms have been proposed, as these hold greater promise than single-agent therapies in addressing thrombotic complications associated with COVID-19^3^. Extracorporeal blood purification has been reported as an effective therapeutic approach to remove excess cytokines during sepsis^4^. This technique is also being investigated on a global scale for its potential to limit severe COVID-19 disease^5^. Additionally, continuous renal replacement therapy is necessary in cases of advanced organ damage, such as advanced acute kidney injury requiring dialysis^6^.

Direct hemoperfusion (DHP) using a polymyxin B-immobilized fiber column (PMX) (Toraymyxin; PMX-DHP) removes endotoxins from the patient blood, using the binding affinity of polymyxin B, a polypeptide antibiotic, to lipid A, the active center of the endotoxin. PMX-DHP is indicated for severe conditions that may be caused by endotoxemia or gram-negative rod infection and is effective not only in sepsis but also in respiratory diseases^7^. The mechanism of PMX therapy for severe pneumonia of nonbacterial origin is unclear. Suggested mechanisms include adsorption and removal of endotoxins, mediators such as inflammatory cytokines involved in cytokine storms, and cellular components such as activated leukocytes that directly injure lung tissue. A genetic overlap between idiopathic pulmonary fibrosis (IPF) and severe COVID-19 has been observed based on large-scale genome-wide association studies^8^. Previously, PMX-DHP has been reported to have therapeutic effects, such as improved lung oxygenation for acute exacerbation of interstitial pneumonia, including IPF manifesting as diffuse alveolar damage^9,10^. Furthermore, early PMX-DHP intervention for acute exacerbations of IPF has been reported to suppress fibrosis progression regardless of the disease stage, and its effectiveness against COVID-19 is also anticipated^11^.

We previously reported our experience with PMX-DHP in 12 patients early in the COVID-19 epidemic^12^ Several observational studies, including ours, have examined the effect of PMX-DHP on COVID-19^13–15^ but no prospective cohort studies have yet been reported. In this study, we conducted a multicenter prospective study to access treatment options to prevent worsening of COVID-19-associated pneumonia.

## Methods

### Study design

We conducted a multicenter, prospective, interventional, single-arm study to evaluate the efficacy and safety of PMX-DHP in patients with COVID-19^16^Because the PMX-DHP performed in this study is a relatively invasive treatment, it is difficult to establish a sham group from an ethical standpoint. Therefore, this was a single-arm study, and the outcomes were compared with those of a synthetic control to evaluate the efficacy of PMX-DHP in patients with COVID-19. For the synthetic control group, we utilized the COVID-19 Registry Japan (COVIREGI-JP) data, which is the largest registry of hospitalized patients with COVID-19 in Japan^17^. The registered patients were diagnosed with COVID-19 and hospitalized at medical institutions in Japan. The matching algorithm for constructing the synthetic control is described in the “Statistical Analysis” section.

### Patient population

This study included patients who had positive nasal swabs for COVID-19, analyzed with real-time, reverse transcriptase-polymerase chain reaction (RT-PCR), who were admitted to the National Center for Global Health and Medicine (NCGM, Tokyo, Japan), and the Tokyo Medical University Hospital (Tokyo, Japan) between September 28, 2020 and March 31, 2022.

### Inclusion/exclusion criteria

The inclusion criteria for the participants were as follows: (1) presence of respiratory distress that cannot be explained by other conditions (such as heart failure or renal failure), (2) confirmed diagnosis of severe acute respiratory syndrome coronavirus 2 infection by RT-PCR or reverse transcription loop-mediated isothermal amplification within the previous week, (3) presence of pneumonia findings on chest imaging, (4) PaO_2_/FiO_2_ (P/F) ratio of 300 or less or peripheral capillary oxygen saturation (SpO_2_) of 93% or less (room air), (5) categorization into grades 4, 5, or 6 according to an 8-grade evaluation, 6) aged 16 years or older at the time of obtaining consent, (7) written consent obtained from the individual or their proxy.

The exclusion criteria were the following: (1) severe progression of multiorgan failure, (2) P/F ratio of 100 or less, (3) undergoing extracorporeal membrane oxygenation (ECMO), (4) hospitalization for more than 15 days, (5) platelet count below 20,000/µL, (6) recent receipt (within 4 weeks prior to consent) of cytotoxic or biological therapies (anti-interleukin-1 [IL-1], anti-IL-6 [tocilizumab or sarilumab], T-cell or B-cell targeted therapy [rituximab, etc.], tyrosine kinase inhibitors, or interferon), (7) administration of tumor necrosis factor (TNF) inhibitors within 2 weeks prior to consent, (8) receipt of convalescent plasma or intravenous immunoglobulin (IVIg) for COVID-19, (9) individuals deemed inappropriate for inclusion in the study by the principal investigator or co-investigators.

### Study definitions and measurements

Efficacy was evaluated based on our previous study of COVID-19 treatment, with a rate of improvement of at least 1 point from an Ordinal Scale for Clinical Improvement of 4, 5, or 6 on Day 15 after PMX-DHP as the primary endpoint^18^ The Ordinal Scale is as follows: (1) no hospitalization and resumption of normal activities; (2) no hospitalization but no resumption of normal activities; (3) hospitalization without a requirement for O_2_ supplementation; (4) hospitalization requiring O_2_ supplementation; (5) hospitalization requiring nasal high-flow O_2_ therapy, noninvasive mechanical ventilation, or both; (6) requiring invasive mechanical ventilation; (7) requiring ventilation and ECMO; and (8) death. Secondary endpoints included worsening status or disease condition and improvement in disease condition. Worsening status was defined as worsening of the Ordinal Scale score by at least one point from baseline on Day 15. Improvement in disease condition was defined on the Ordinal Scale of 1, 2, or 3 on Day 15. Worsening disease conditions were defined on the Ordinal Scale of 5, 6, 7, or 8 on Day 15, excluding those who improved on their Ordinal Scale. Safety was assessed based on the incidence of serious adverse events^17^.

### Clinical procedure

For PMX-DHP, a temporary blood access catheter was inserted and extracorporeal circulation was established. PMX-DHP utilized Toraymyxin PMX-20R (Toray Industries, Inc., Tokyo, Japan) at a blood flow rate of 60–120 mL/min, administered for a maximum duration of 3 h over 2–3 consecutive days. The circuit underwent reassembly before each treatment session. Nafamostat mesylate (30–40 mg/h) or 40–50 U/kg/h low-molecular-weight heparin was used as anticoagulation therapy. In the event of circuit coagulation, a new circuit was primed and re-established after the blood was returned.

Heparin or nafamostat mesylate was used for anticoagulation therapy as a part of hemodialysis in conjunction with PMX therapy, as indicated in the package insert. As patients eligible for this treatment were within the range of heparin and nafamostat mesylate levels, this treatment was not considered part of the study.

No restrictions were imposed on the antiviral therapy for COVID-19; however, as a general rule, no changes in antiviral therapy were made during the study period. Biological therapies targeting cytokines (anti-IL-1, anti-IL-6 [sarilumab], T- or B-cell-targeted therapy [such as rituximab], tyrosine kinase inhibitors, or interferon), TNF inhibitors, convalescent plasma, or IVIg for COVID-19 were not allowed. The concomitant use of steroids, tocilizumab, and baricitinib was permitted.

Corticosteroid dosage was determined by the attending physician, and options such as continuous infusion at a rate of 10 mg/h following the administration of 200 mg hydrocortisone, steroid pulse therapy (methylprednisolone 500–1000 mg/day for three days), and daily administration of 6 mg dexamethasone were chosen. The tapering regimen for corticosteroids was determined by the attending physician.

### Measurements

Urinary β2-microglobulin and liver-type fatty acid-binding protein (L-FABP) levels were determined using latex-enhanced turbidimetric immunoassays (Denka Seiken and Sekisui Medical, Tokyo, Japan)^19,20^ Patient serum IL-6 levels were analyzed using a Bio-Plex suspension assay kit and array system (BioRad Laboratories, CA, USA) according to the manufacturer’s instructions. Bioplex analysis of serum samples was conducted at the National Center for Global Health and Medicine.

### Electron microscopy

After treatment, the columns were perfused with an aldehyde mixture (2% paraformaldehyde and 2% glutaraldehyde in 30 mM 4-(2-Hydroxyethyl)-1-piperazineethanesulfonic acid (HEPES) buffer containing 100 mM NaCl and 2 mM CaCl2, pH adjusted to 7.4) and prefixed for 1 h at room temperature. The cells were post-fixed in an aldehyde-OsO_4_ mixture (1% OsO_4_, 1.25% glutaraldehyde, 1% paraformaldehyde, and 0.32% K_3_[Fe(CN)_6_]) in 30 mM HEPES buffer (pH 7.4) for 1 h at room temperature. The fixed samples were washed twice with Elix water (Merck Millipore, Burlington, MA, USA) and dehydrated using a graded ethanol series. The samples were analyzed by scanning and transmission electron microscopies (SEM and TEM). For SEM, dehydrated samples were immersed twice for 10 min in pure hexamethyldisilazane followed by 15 min of air-drying, and sputter-coated with platinum using a magnetron sputter (MSP-1S; Vacuum Device, Tokyo, Japan)^21^. Samples were examined under a scanning electron microscope (IT300; JEOL, Tokyo, Japan). For TEM, dehydrated samples were infiltrated with propylene oxide and embedded in epoxy resin (Quetol 651; Nisshin EM Co. Ltd., Tokyo, Japan). Resin blocks were sectioned at 80 nm thicknesses using an ultramicrotome (Leica EM UC7; Leica, Wetzlar, Germany), contrasted with uranyl acetate and lead citrate. Sections were examined under a transmission electron microscope (JEM-1400; JEOL, Tokyo, Japan)^22^.

### Statistical analysis

This single-arm study was designed to enroll 30 patients, expecting the improvement rate of the PMX-DHP group to be 1.5 times that of the synthetic control group. If the improvement rate of the synthetic control group was 55% and 90 patients were extracted for the synthetic control group, there would be 83% power to detect the difference between the two groups under a 10% two-sided significance level.

The full analysis set (FAS) was defined as the set of all participants enrolled in the study, excluding those who never received any protocol treatment, those who received at least one protocol treatment but for whom no post-treatment data were available, and those who were ineligible. We conducted the following steps to generate a synthetic control group by matching the COVIREGI-JP participants with the FAS participants. First, we extracted participants registered in the COVIREGI-JP according to the inclusion and exclusion criteria of the study. Second, the synthetic control participants were selected via exact matching of the following variables measured at baseline: the same 8-category status on Study Day 1 or 2, the same SpO_2_ level (above or below 93%), and the time of hospitalization belonging to the same epidemic wave in Japan. Finally, among the selected participants by exact matching, at most five (1:1 to 5:1) synthetic control participants with the closest propensity scores within the prespecified caliper (0.2 times the standard deviation of the logit-transformed value for the propensity score in the FAS) were matched to each FAS participant. The propensity score for each individual was estimated using a logistic regression model, including the following: age, sex, body mass index, vaccination status, D-dimer, C-reactive protein, fraction of inspired oxygen, and SpO_2_. The participants in the synthetic control group were weighted using reciprocal numbers of synthetic control participants in their matched sets (weights ranged from 0.2 to 1) in each analysis.

Baseline characteristics were presented with mean, standard deviation, median, and range for continuous variables and counts and percentages for categorical variables. Standardized differences were computed for baseline variables. Differences between the PMX-DHP group and the synthetic control group in primary and secondary efficacy endpoints were assessed using risk ratios and corresponding 95% confidence intervals (CIs) and compared using the chi-squared test. Kaplan–Meier curves for time to death were estimated and compared using the log-rank test. Statistical significance was set at *p* < 0.1 for the primary endpoint and *p* < 0.05 for the other endpoints. We performed all the statistical analyses using SAS version 9.4 (SAS Institute, Cary, NC, USA).

### Ethics approval

This study was conducted in accordance with the Declaration of Helsinki and the Clinical Trials Act (Act No. 16 of April 14, 2017). The Certified Review Board of the National Center for Global Health and Medicine approved the study protocol for clinical research (approval no: NCGM-C-003585-06, CRB3200011), and written informed consent for publication was obtained from each patient.

## Results

### Study flow

**Figure 1:**
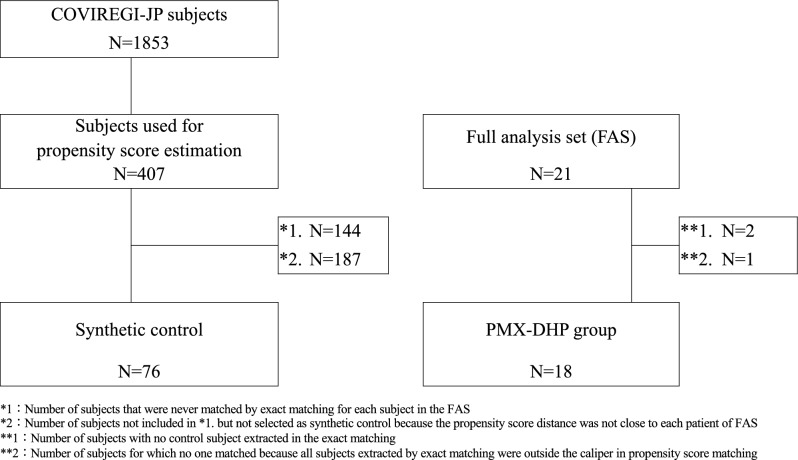
Flow diagram of the study. A total of 21 patients received PMX-DHP therapy in this study; 3 patients were excluded for reasons related to matching in the control group. In the control group, 1853 patients were selected from the COVIREGI-JP database, of which 407 were used for propensity score estimation. Finally, 76 individuals were selected as synthetic controls. COVIREGI-JP, COVID-19 Registry Japan; PMX-DHP, polymyxin B immobilized fiber column direct hemoperfusion.

Figure 1 shows a flow diagram of the study. A total of 21 patients received PMX-DHP therapy in this study. Three were excluded because of reasons related to the matching of the control group. In the control group, 1853 individuals were extracted from the COVIREGI-JP database, of which 407 were used for propensity score estimation. Finally, 76 participants were selected as synthetic controls, and a control group equivalent to 18 participants was established after weighing up to 5:1 as described in the “Methods” section.

### Patients backgrounds

Patient characteristics are shown in Table 1. Most patients were male, with a median age of 54.5 years in the PMX group and 59.0 years in the control group. The duration from symptom onset was approximately 10 d in both groups. Regarding the 8-category status at enrollment, the proportion of intubated patients was approximately 10% in both groups; however, the PMX group showed a higher percentage of patients receiving high-flow oxygen therapy and non-invasive mechanical ventilation (22.2 vs. 10.0%).

**Table 1:** Baseline characteristics.

		Full analysis set (N = 21)	PMX-DHP group (N = 18)	Synthetic control (N = 18)	Standardized difference*
Sex	Male	18 (85.7)	15 (83.3)	16.0 (88.9)	− 0.16
Age	Mean (SD)	58.1 (11.2)	57.4 (11.7)	60.9 (18.2)	− 0.23
	Median (range)	55.0 (40–79)	54.5 (40–79)	59.0 (25–100)	
BMI	Missing	6	5	3.3	− 0.38
	Mean (SD)	25.4 (3.4)	25.0 (3.1)	26.7 (5.3)	
	Median (range)	25.0 (19.8–31.5)	25.0 (19.8–30.0)	26.1 (16.8–47.5)	
Days from onset	Mean (SD)	9.9 (3.0)	10.4 (2.3)	10.3 (3.8)	0.03
	Median (range)	10.0 (3–15)	10.5 (6–15)	10.0 (3–27)	
Vaccination history	Vaccinated	3 (14.3)	0 (0.0)	1.0 (5.6)	0.34
Number of comorbidities	Mean (SD)	1.0 (0.9)	0.9 (0.9)	1.3 (1.2)	− 0.30
	Median (range)	1.0 (0–3)	1.0 (0–3)	1.0 (0–4)	
SpO_2_ (%)	Mean (SD)	90.9 (3.2)	91.0 (3.5)	91.2 (6.1)	− 0.05
	Median (range)	91.0 (80–97)	91.5 (80–97)	93.0 (68–98)	
FiO_2_ (%)	Missing	0	0	2.1	− 0.26
	Mean (SD)	47.3 (15.9)	44.8 (15.7)	50.4 (25.9)	
	Median (range)	44.0 (24.0–71.7)	40.3 (24.0–71.7)	40.0 (5.0–98.0)	
D-dimer (μg/mL)	Missing	0	0	1.4	0.16
	Mean (SD)	4.2 (7.9)	4.3 (8.5)	2.8 (9.4)	
	Median (range)	1.4 (0.0–33.5)	1.1 (0.0–33.5)	1.0 (0.0–80.6)	
CRP (mg/dL)	Missing	0	0	1.4	− 0.21
	Mean (SD)	7.8 (7.1)	8.0 (7.7)	12.7 (8.9)	
	Median (range)	5.7 (1.6–34.4)	5.5 (1.6–34.4)	10.4 (0.3–30.7)	
LDH (U/L)	Missing	0	0	2.2	− 0.03
	Mean (SD)	446.4 (185.7)	439.6 (189.4)	445.2 (176.7)	
	Median (range)	372.0 (260–858)	369.5 (260–858)	387.0 (192–1112)	
8-category status at baseline	4	12 (57.1)	12 (66.7)	14.2 (78.9)	− 0.28
	5	7 (33.3)	4 (22.2)	1.8 (10.0)	0.34
	6	2 (9.5)	2 (11.1)	2.0 (11.1)	0.00
8-category status at day 15	1	6 (28.6)	6 (33.3)	9.1 (50.6)	
	2	0 (0.0)	0 (0.0)	0.4 (2.2)	
	3	6 (28.6)	5 (27.8)	2.0 (11.1)	
	4	5 (23.8)	4 (22.2)	1.9 (10.6)	
	5	2 (9.5)	1 (5.6)	1.7 (9.4)	
	6	2 (9.5)	2 (11.1)	1.5 (8.3)	
	7	0 (0.0)	0 (0.0)	0.0 (0.0)	
	8	0 (0.0)	0 (0.0)	1.4 (7.8)	

*SD* standard deviation. The 8-category status is as follows: (1) no hospitalization and resumption of normal activities; (2) no hospitalization but no resumption of normal activities; (3) hospitalization without a requirement for O_2_ supplementation; (4) hospitalization requiring O_2_ supplementation; (5) hospitalization requiring nasal high-flow O_2_ therapy, noninvasive mechanical ventilation, or both; (6) requirement for invasive mechanical ventilation; (7) requirement for a ventilator and extracorporeal membrane oxygenation (ECMO); and (8) death. If any missing values occur, present the number of missing values. *Standardized difference between PMX-DHP group and Synthetic control group. *BMI* body mass index, *CRP* C-reactive protein, *LDH* lactate dehydrogenase.

Concomitant medications used during hospitalization are listed in Supplementary Table 1. Remdesivir was administered to 83 and 92% of patients in the PMX and control groups, respectively. Corticosteroids were used in all patients in both groups, and a higher tendency to use high doses of methylprednisolone (250 mg/day or more) was observed in the PMX group.

### Primary and secondary endpoints

**Figure 2:**
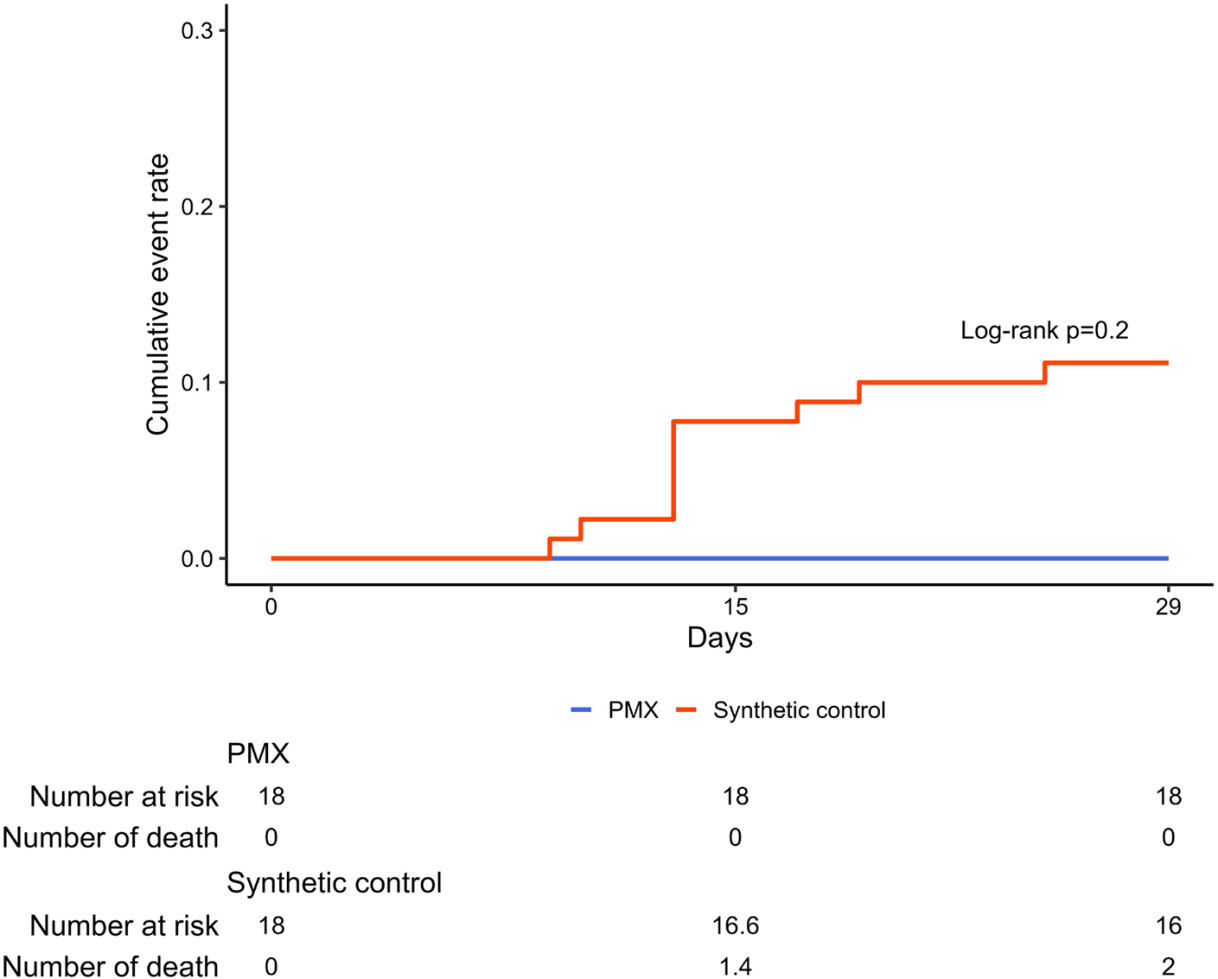
Kaplan–Meier estimate. Mortality on Day 15 was 0% in the PMX-treated group and 7.8% in the control group; mortality on Day 29 was 0% in the PMX-treated group and 11.1% in the control group.

The primary endpoint (percentage status improvement on Day 15) did not differ between the PMX-treated and control groups (Table 2). The risk ratio was estimated as 1.0 and corresponding 90 and 95% CIs were (0.68–1.47) and (0.63–1.59), respectively, and the percentage of status improvement was 66.7% (12/18) in both the PMX-treated and control groups. The secondary endpoint, the percentage with worsening status on Day 15, was 5.6% in the PMX-treated group and 14.4% in the control group, indicating that the PMX-treated group was 0.38 times (95% CI 0.04–3.51) more likely to prevent status deterioration. In terms of the percentage of patients requiring intensive care on Day 15, 16.7% of PMX-treated patients and 22.8% of control patients were worse, indicating that the PMX-treated group was 0.73 times (95% CI 0.19–2.79) more likely to reduce demand for intensive care (Table 2). The mortality rates on Day 29 were 0% (0/18) in the PMX-treated group and 11.1% (2/18) in the control group (Fig. 2). We reviewed the cause of death of the patients who died in the control group. We found that most of the patients were elderly and obese, all had acute respiratory distress syndrome exacerbated by COVID-19, and all had respiratory failure as the cause of death. Furthermore, we investigated the patient demographics within the PMX treatment cohort, distinguishing between those who exhibited improvement and those who did not (Supplementary Table 2). Eight of the 12 patients (66.7%) who required low-flow oxygen (severity status 4) at the start of PMX improved. Of the seven patients who required high-flow oxygen (severity status 5), six (85.7%) improved. In contrast, none of the severity status 6 patients who required intubation and ventilator management at the start of PMX improved.

**Table 2:** Efficacy endpoints.

	# of events of PMX-DHP group (percent)	# of events of synthetic control (percent)	Risk ratio	95% CI	*p* value of chi-squared test
Improving status	12 (66.7)	12.00 (66.7)	1.00	[0.63–1.59]	1.000
Worsening status	1 (5.6)	2.60 (14.4)	0.38	[0.04–3.51]	0.374
Improvement in disease condition	11 (61.1)	11.50 (63.9)	0.96	[0.58–1.59]	0.863
Worsening disease condition	3 (16.7)	4.10 (22.8)	0.73	[0.19–2.79]	0.645

Improving status was defined as an improvement of Ordinal Scale by at least one point at Day 15 from baseline. Worsening status was defined as a worsening of Ordinal Scale by at least one point at Day 15 from baseline. Improvement in disease condition was defined as Ordinal Scale of 1, 2, or 3 at Day 15. Worsening disease condition was defined as an Ordinal Scale score of 5, 6, 7, or 8 at Day 15, excluding those who improve their Ordinal Scale.

### The clinical efficacy of the PMX treatment group

**Figure 3:**
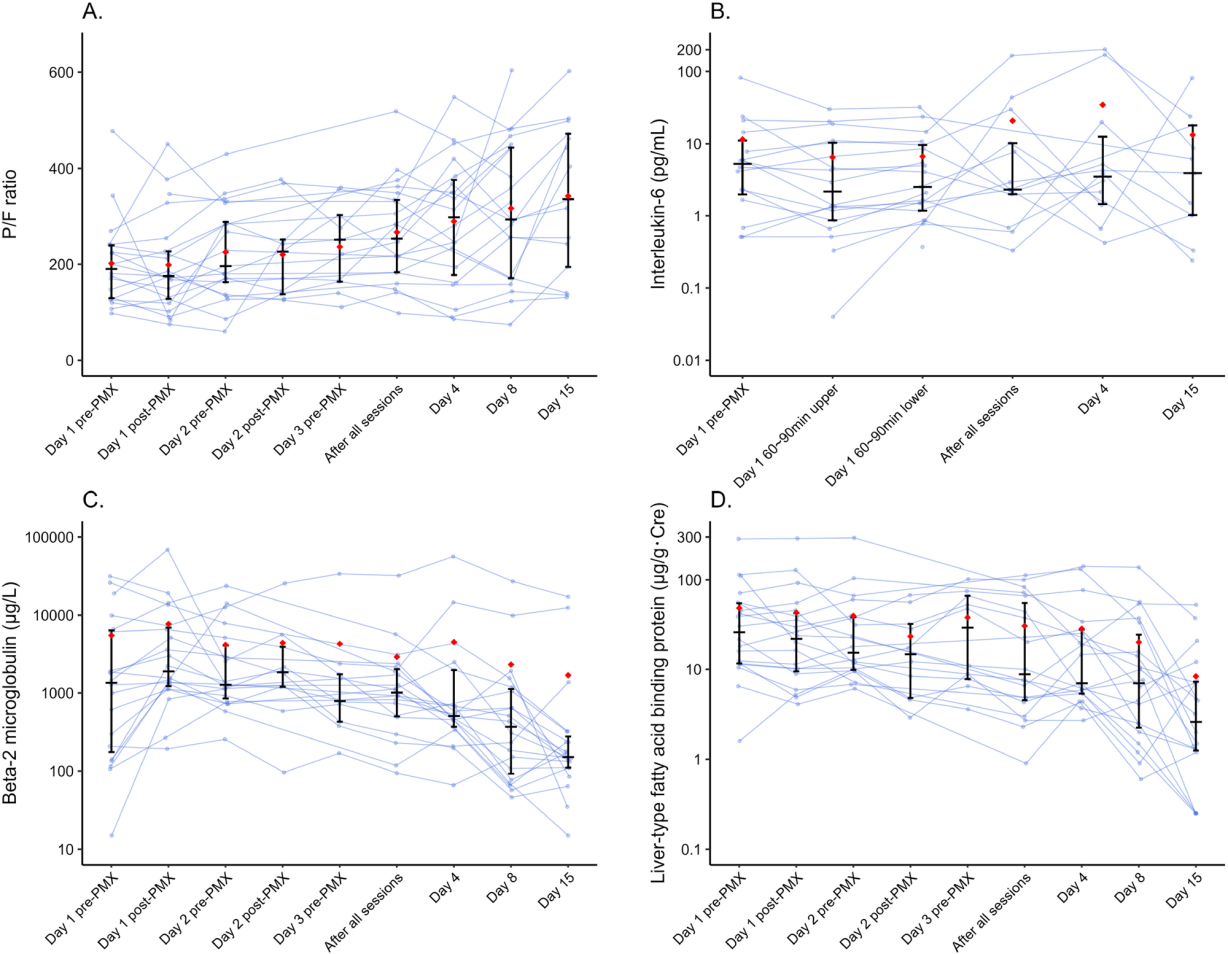
Clinical effectiveness in the PMX-treated group. Variations in various parameters before, during, and after PMX treatment for FAS. Horizontal black bars indicate the first quartile, median, and third quartile at each time point. Red diamonds indicate mean values at each time point. (**A**) P/F ratio, (**B**) serum IL-6, (**C**) urinary β2-microbloburin, (**D**) urinary L-FABP. L-FABP, liver-type fatty acid-binding protein; P/F ratio, PaO_2_/FiO_2_ ratio.

P/F ratios before and after PMX treatment are shown in Fig. 3A. The P/F ratio improved before and after treatment (before treatment, 201.9 ± 90.0; Day 4, 289.0 ± 131.8, and Day 8, 317.0 ± 144.7). IL-6 decreased once during PMX treatment, but this decrease did not persist (Fig. 3B; Before treatment, 11.5 ± 20.1; during treatment, 6.5 ± 8.6; Day 4, 34.6 ± 71.3, and Day 15, 13.3 ± 23.8). Urinary biomarkers showed a marked decrease in both β2-microglobulin and L-FABP before and after treatment (Fig. 3C; β2-microglobulin; before treatment; 5505.6 ± 9244.2; Day 4, 4516.7 ± 12,989.7, and Day 15, 1680.3 ± 4566.8, Fig. 3D; L-FABP; before treatment, 48.6 ± 64.5; Day 4, 28.6 ± 42.1, and Day 15, 8.4 ± 13.8). Serious adverse events associated with PMX treatment included respiratory failure (2/21, 9.5%). Problems that could not be ruled out as causally related to PMX treatment included hematochezia in one patient (1/21, 4.8%), prolonged activated partial thromboplastin time in two patients (2/21, 9.5%), and blood in the urine of one patient (1/21, 4.8%). There were no events such as blood pressure fluctuations or catheter-related infections during PMX treatment. In the PMX group, the inlet pressure increased in 11/53 sessions (20.8%), circuit coagulation and membrane exchange occurred in 2/53 sessions (3.8%), respectively. Of these, circuit coagulation led to membrane replacement in 1/53 (1.9%) sessions.

After the first PMX-DHP treatment, the columns were examined using electron microscopy (Supplementary Fig. S1). The cells were shown to be tightly attached to the PMX fibers. Despite the presence of numerous white blood cells, many areas where fibrin formed by platelet clumps and red blood cells were noted. Moreover, leukocytes were variably deformed and actively clung to the porous PMX fibers by extending their pseudopodia. 

## Discussion

Treatment modalities that can nonspecifically control disease progression until a specific drug or vaccine is developed, are not only imperative for H1N1 influenza^7^ and COVID-19 but also for future possible outbreaks.

Hemoperfusion has a simple and closed-circuit configuration. Still, it requires attention to the use of anticoagulants and biocompatibility of the membrane because of the direct contact between the blood and the sorbent. Hemoperfusion devices are classified as selective (e.g., toraymyxin) or nonselective (e.g., CytoSorb), each with a different therapeutic target. The results of this multicenter prospective study suggest that it may be useful to consider administration in patients with a P/F ratio < 300 or moderate disease II or higher requiring oxygen administration, but before the patient becomes severe enough to require ventilatory management or ECMO. We conducted an endotoxin activity assay (EAA) on select patients within the PMX treatment group. The results encompassed pre-treatment values (0.34 ± 0.14, mean ± standard deviation) and post-treatment values (0.35 ± 0.22). Consequently, our investigation focused on evaluating the impact of PMX-DHP treatment on a subset of patients exhibiting oxygen demand despite relatively low EAA levels. De Rosa et al. administered PMX-DHP in patients with COVID-19 experiencing endotoxemia from the Early Use of Polymyxin B Hemoperfusion in Abdominal Septic Shock 2 (EUPHAS2) registry^13^ The study reported an improvement in the Sequential Organ Failure Assessment (SOFA) score and a reduction in EAA at 120 h after two 2-h sessions of PMX-DHP in 12 patients who required ventilator support. Peerapornratana et al. performed PMX-DHP in several COVID-19 patients and reported reductions in IL-6 and EAA^23^

This was a single-arm, open-label study and the first English-language study to evaluate the efficacy of PMX-DHP in patients with COVID-19 by comparing the results with those of a synthetic control group. The synthetic control group used data from the largest COVID-19 inpatient registry in Japan. The medical response to COVID-19 has led to a search for new treatments, which posed several challenges, such as the importance of selecting the appropriate target and timing of treatment. The present study showed that PMX treatment effectively prevented the worsening of COVID-19 pathology. However, IL-6 levels were not persistently reduced. An observational study on PMX-DHP for COVID-19 in Japan^15^ also showed no effect on IL-6 levels. It is widely known that IL-6 levels are lower in patients with COVID-19 than in patients with sepsis or acute respiratory distress syndrome^24^ and the effectiveness of treatment targeting IL-6 alone is questionable. For example, a randomized controlled trial (RCT) of patients with severe COVID-19 who were referred for ECMO reported that the Cytosorb (CytoSorbents, NJ, USA) treatment group not only failed to reduce IL-6 but also had a significantly higher 30-day mortality rate^25^ Questions regarding randomization, timing of ECMO, and serum IL-6 levels have been raised, and no conclusions can be drawn. Additionally, it is recognized that severe COVID-19 cases often entail activation of blood coagulation and vascular endothelial damage due to inflammation. It is proposed that the removal of activated leukocytes, platelets, and fibrin through acute blood purification therapy, as observed by electron microscopy in this study, could potentially forestall the subsequent advancement toward multiorgan failure. However, for PMX-DHP, the selection and timing of treatment targets for COVID-19 are also important. A multicenter RCT for endotoxemia, the EUPHRATES study^26^ was published in 2018. The 28-day mortality rates were 37.7 and 34.5% in the PMX and sham groups, respectively, which were not significantly different. However, subsequent subgroup analysis^27^ showed a 10.7% reduction in mortality. In a study on COVID-19 by Kuwana et al., the parameters before PMX-DHP that differed significantly between the survival and death groups were the rate of invasive ventilation, P/F ratio, and SOFA score^28^ suggesting that the selection of appropriate patients and the timing of the procedure are important in COVID-19 as well^29^

We have reported that the noninvasive measurement of urinary β2-microglobulin or urinary L-FABP is related to the severity of COVID-19 and can be a useful predictive tool^30,31^ β2-microglobulin is elevated in inflammatory and cytokine-overload conditions, and L-FABP is elevated in hypoxia under inflammatory and cytokine-rich conditions. PMX treatment markedly reduced L-FABP and increased the P/F ratio, suggesting that PMX may positively affect moderately to severely hypoxic patients with COVID-19 exhibiting progressive hypoxia. In our previous multicenter study, we used urine L-FABP in COVID-19 to predict serious outcomes^32^ The area under the curve of L-FABP for predicting serious outcomes, including ventilator placement, was 96.3% (95% CI 92.6–98.8%), with a cutoff of 35.9 μg/gCre. Sensitivity and specificity were 100 and 96.3%, respectively. In the present study, the pre-procedure L-FABP in the PMX group was 48.6 (11.7–55.2) μg/gCre (Fig. 3D), which would have been expected to be a severe outcome in the natural course. Electron microscopic examination of the columns revealed that fibrin produced by granulocytes and platelets adhered to the membrane in many erythrocytes. The possibility that removing these activated cells by PMX treatment may be beneficial in improving the condition has been reported in acute exacerbations of interstitial pneumonia^33^ and a similar effect can be expected in patients with COVID-19. In addition, post-treatment electron microscopic findings of numerous activated white blood cells, clumps of platelets, and red blood cells attached to fibrin suggest that the removal of microthrombi may be part of the therapeutic effect.

One limitation of this study is the problem associated with using historical controls. Although baseline matching was performed, expecting a perfect match of backgrounds is impossible^34^ In addition, the data for the historical control group utilizes the COVID-19 registry, and Day 1 and Day 4 are purely days from admission, different from the PMX treatment group. Also, participants in the historical control group had more missing data than those in the treatment group, which means that caution should be exercised in interpreting the results. The constraints within the registry precluded the computation of the SOFA, Acute Physiology and Chronic Health Evaluation, or Simplified Acute Physiology 2 scores. Moreover, due to the scarcity of medical resources and the challenge of controlling COVID-19 infections during a pandemic, quantifying activated cells before and after PMX treatment proved unattainable in this study. Consequently, membranes were directly examined via electron microscopy post-treatment. The possibility of future infectious disease pandemics is well established. Initially, in phases where vaccines and therapeutic agents are not fully developed, it has been suggested that blood purification therapy may contribute to the control of the progression and preventing the worsening of the patient’s condition.

In conclusion, PMX-DHP was shown to be potentially effective in preventing the worsening of oxygenation capacity and disease progression in patients with COVID-19-associated pneumonia who require oxygen.

### Supplementary Information

Supplementary Information 1.

Supplementary Information 2.

Supplementary Information 3.

## Data Availability

The data obtained at NCGM in this study will be registered in the “Registry research relating to COVID-19 (COVIREGI) (NCGM Ethical Inspection Committee approval No. NCGM-S-003494–41)”. The data for this study are available from the corresponding author, Shinyu Izumi, upon reasonable request.
