# Supplementary material for: Blood perfusion with polymyxin B immobilized columns in patients with COVID-19 requiring oxygen therapy
Source: Sci Rep. 2024 May 31;14:12550. doi: 10.1038/s41598-024-63330-2 (PMC11143350; doi:10.1038/s41598-024-63330-2)
Supplement: Supplementary file 1 — Supplementary Information 1. [file 41598_2024_63330_MOESM1_ESM.pdf]

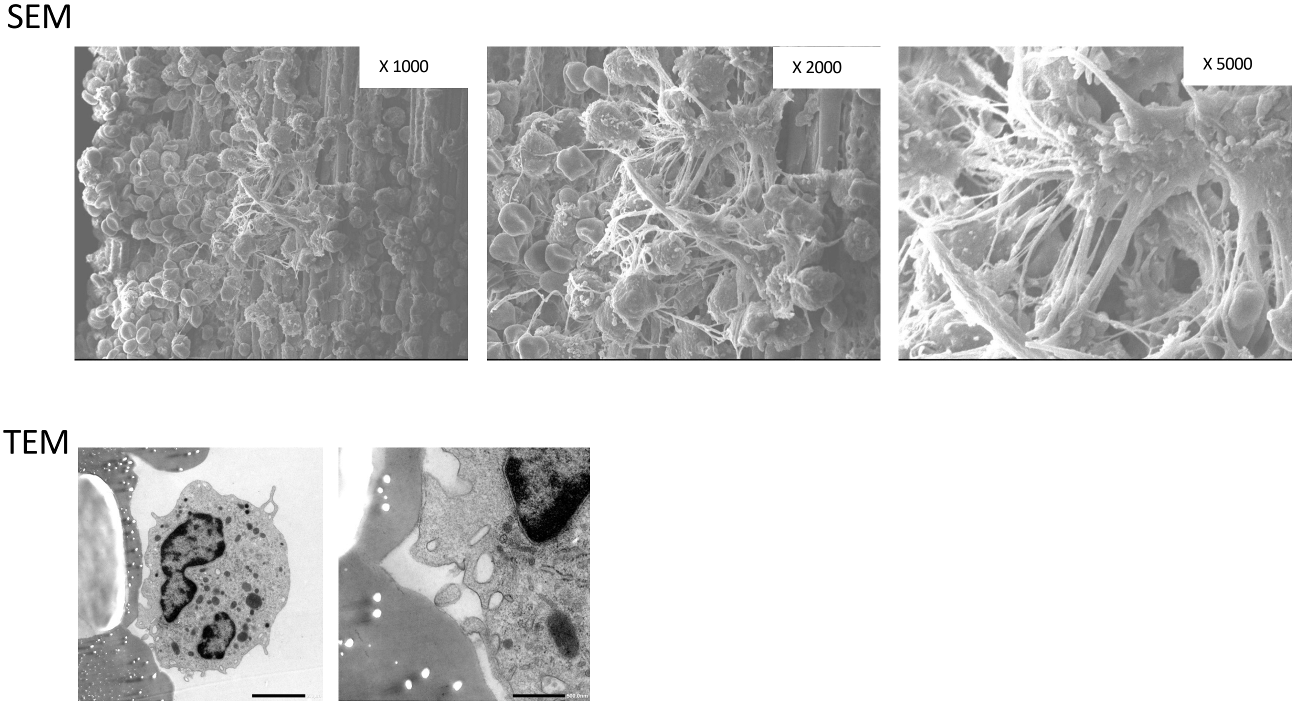

Supplementary Figure S1. Electron microscopic findings after PMX treatment.
Numerous activated white blood cells, clumps of platelets and red blood cells adhering to fibrin can be seen. In addition, the white blood cells were variously deformed and actively adhering to the porous PMX fibers with pseudopodia extension. TEM showed that most of these adsorbed cells were neutrophils. SEM; Scanning electron microscope, TEM; Transmission electron microscope.
