# Supplementary material for: Blood perfusion with polymyxin B immobilized columns in patients with COVID-19 requiring oxygen therapy
Source: Sci Rep. 2024 May 31;14:12550. doi: 10.1038/s41598-024-63330-2 (PMC11143350; doi:10.1038/s41598-024-63330-2)
Supplement: Supplementary file 2 — Supplementary Information 2. [file 41598_2024_63330_MOESM2_ESM.docx]

|  |  | PMX-DHP group (N=18) | Synthetic control (N=18) |
| --- | --- | --- | --- |
|  |  |  |  |
| Remdesivir | Not used | 3 (16.7) | 0.4 (2.2) |
|  | Used | 15 (83.3) | 16.6 (92.2) |
|  | Unknown | 0 (0.0) | 1.0 (5.6) |
| Tocilizumab | Not used | 16 (88.9) | 13.7 (76.1) |
|  | Used | 2 (11.1) | 3.3 (18.3) |
|  | Unknown | 0 (0.0) | 1.0 (5.6) |
| Baricitinib | Not used | 13 (72.2) | 14.6 (81.1) |
|  | Used | 5 (27.8) | 2.4 (13.3) |
|  | Unknown | 0 (0.0) | 1.0 (5.6) |
| Steroid | Not used | 0.0 (0.0) | 0.0 (0.0) |
|  | Used | 18 (100.0) | 18 (100.0) |
| Maximum dose of steroid | Methylprednisolone ≥250mg/day and ≤1000mg/day (or equivalent dose) | 10 (55.6) | 5.2 (28.9) |
|  | Methylprednisolone ≥60mg/day and <250mg/day (or equivalent dose) | 2 (11.1) | 4.5 (25.0) |
|  | Methylprednisolone <60mg/day (or equivalent dose) | 1 (5.6) | 7.5 (41.7) |
|  | Dexamethasone 6.0-6.6mg/day | 5 (27.8) | 0.8 (4.4) |

Supplementary table 1 Concomitant drugs used during hospitalization
