# Supplementary material for: Blood perfusion with polymyxin B immobilized columns in patients with COVID-19 requiring oxygen therapy
Source: Sci Rep. 2024 May 31;14:12550. doi: 10.1038/s41598-024-63330-2 (PMC11143350; doi:10.1038/s41598-024-63330-2)
Supplement: Supplementary file 3 — Supplementary Information 3. [file 41598_2024_63330_MOESM3_ESM.docx]

|  | | Improved patients  (N=14) | Not improved patients  (N=7) |
| --- | --- | --- | --- |
|  |  |  |  |
| Sex | Male | 13 (92.9) | 5 (71.4) |
| Age | mean (SD) | 53.5 (7.4) | 67.3 (12.2) |
|  | median (range) | 52.5 (40.0 - 67.0) | 72.0 (44.0 - 79.0) |
| BMI | missing | 5 | 1 |
|  | mean (SD) | 25.6 (3.7) | 25.1 (3.1) |
|  | median (range) | 26.4 (19.8 - 31.5) | 25.0 (20.4 - 30.0) |
| Days from onset | mean (SD) | 9.0 (3.1) | 11.6 (2.1) |
|  | median (range) | 10.0 (3.0 - 13.0) | 11.0 (9.0 - 15.0) |
| Vaccination history | Vaccinated | 2 (14.3) | 1 (14.3) |
| Number of comorbidities | mean (SD) | 0.9 (0.7) | 1.1 (1.3) |
|  | median (range) | 1.0 (0.0 - 2.0) | 1.0 (0.0 - 3.0) |
| SpO2 (%) | mean (SD) | 91.8 (2.2) | 89.1 (4.3) |
|  | median (range) | 92.0 (88.0 - 97.0) | 90.0 (80.0 - 94.0) |
| FiO2 (%) | mean (SD) | 44.0 (15.8) | 54.1 (14.8) |
|  | median (range) | 42.0 (24.0 - 71.7) | 57.5 (36.0 - 70.0) |
| D-dimer (μg/mL) | mean (SD) | 3.3 (4.9) | 5.9 (12.2) |
|  | median (range) | 1.5 (0.0 - 18.4) | 1.1 (0.6 - 33.5) |
| CRP (mg/dL) | mean (SD) | 8.9 (8.5) | 5.8 (2.6) |
|  | median (range) | 6.5 (1.6 - 34.4) | 5.7 (2.4 - 9.3) |
| LDH (U/L) | mean (SD) | 425.1 (178.1) | 488.9 (207.5) |
|  | median (range) | 376.0 (260.0 - 858.0) | 372.0 (273.0 - 793.0) |
| 8-category status at baseline | 4 | 8 (57.1) | 4 (57.1) |
|  | 5 | 6 (42.9) | 1 (14.3) |
|  | 6 | 0 (0.0) | 2 (28.6) |

Supplementary table 2 Baseline Characteristics of improved and not improved patients
